# Ubiquitin C decrement plays a pivotal role in replicative senescence of bone marrow mesenchymal stromal cells

**DOI:** 10.1038/s41419-017-0032-5

**Published:** 2018-01-30

**Authors:** Jiyeon Kim, Yonggoo Kim, Hayoung Choi, Ahlm Kwon, Dong Wook Jekarl, Seungok Lee, Woori Jang, Hyojin Chae, Jung Rok Kim, Jung Min Kim, Myungshin Kim

**Affiliations:** 1 Catholic Genetic Laboratory Center, College of Medicine, The Catholic University of Korea, Seoul, 06591 Republic of Korea; 2 Department of Laboratory Medicine, College of Medicine, The Catholic University of Korea, Seoul, 06591 Republic of Korea; 3 NAR Center, Inc., Daejeon Oriental Hospital of Daejeon University, Daejeon, 34520 Republic of Korea

## Abstract

Human bone marrow-mesenchymal stromal cells (hBM-MSCs) undergo cellular senescence during in vitro culture. In this study, we defined this replicative senescence as impaired proliferation, deterioration in representative cell characteristics, accumulated DNA damage, and decreased telomere length and telomerase activity with or without genomic abnormalities. The *UBC* gene expression gradually decreased during passaging along with the reduction in series of molecules including hub genes; *CDK1, CCNA2, MCM10, E2F1, BRCA1, HIST1H1A* and *HIST1H3B*. *UBC* knockdown in hBM-MSCs induced impaired proliferation in dose-dependent manner and showed replicative senescence-like phenomenon. Gene expression changes after *UBC* knockdown were similar to late passage hBM-MSCs. Additionally, *UBC* overexpession improved the proliferation activity of hBM-MSCs accompanied by increased expression of the hub genes. Consequently, *UBC* worked in higher-order through regulation of the hub genes controlling cell cycle and proliferation. These results indicate that the decrement of *UBC* expression plays a pivotal role in replicative senescence of hBM-MSCs.

## Introduction

Mesenchymal stromal cells (MSCs) are the major cellular component of a niche and reside in virtually all post-natal organs and tissues^1^. They possess a self-renewal capacity and can differentiate into a variety of cell types. Taking advantage of these characteristics, MSCs have been considered as one of the important sources of regenerative medicine. But unlike embryonic stem cells or induced pluripotent stem cells (iPSCs), MSCs lose their proliferation activity and original characteristics after repetitive subculture, although they are considered to have a stemness nature^2^. Aging tissues experience a progressive decline in homeostatic and regenerative capacities, which has been attributed to degenerative changes in tissue-specific stem cells, stem cell niches and systemic cues that regulate stem cell activity^3^. This age-dependent deterioration of stem cell function is thought to be similar to the phenomenon experienced by MSCs after repetitive culture. MSCs undergo only a limited number of cell divisions under standard culture conditions in a process called replicative senescence that results in extensive phenotypic changes and abrogates the in vivo therapeutic potential of MSCs^4^.

Human bone marrow MSCs (hBM-MSCs) are the most investigated source of adult stem cells. Extensive expansion of hBM-MSCs is essential, and it should be performed without change of their original identity^5,6^. For efficient and effective application of hBM-MSCs in regenerative therapy, more evidence and understanding of the replicative senescence of hBM-MSCs are crucial.

With this in mind, we devised the present study to define the characteristics of replicative senescence in hBM-MSCs and to understand the mechanism of impaired proliferation. We thoroughly evaluated the biological and genetic changes during in vitro culture. In parallel, integrative molecular signal network analyses using gene expression data were performed to explain the molecular details of replicative senescence^7^. Eventually, we identified a molecule that is crucial in the impaired proliferation during replicative senescence of hBM-MSCs.

## Results

### Biological characteristics of hBM-MSCs during in vitro culture

**Fig. 1:**
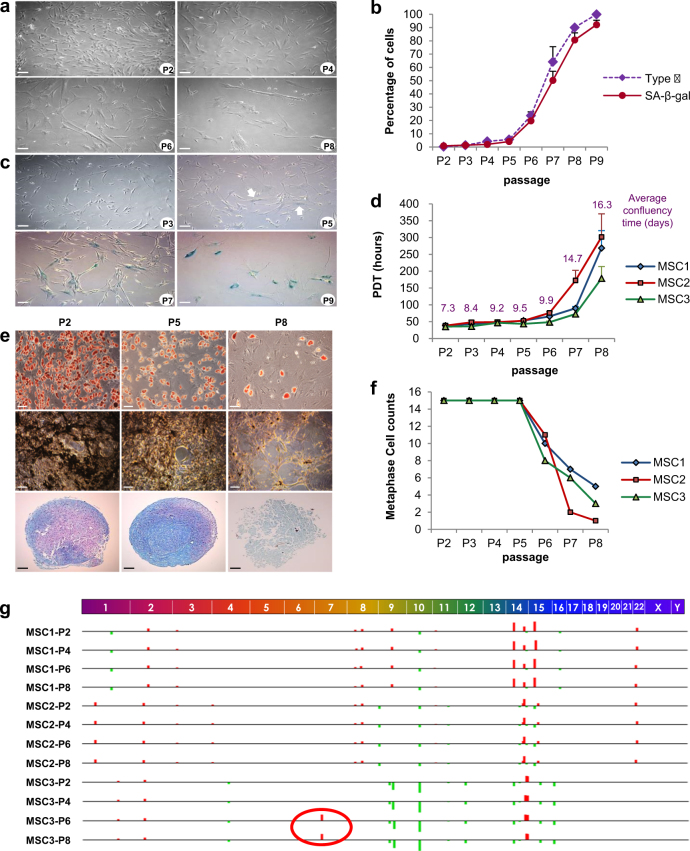
Biological and genetic characteristics of replicative senescence in hBM-MSCs **a** Morphologic changes during in vitro culture. Typical homozygous populations of fibroblast-like cells were observed at P2 and P4. Enlarged type II cells with altered morphology were evident at P6 and were more prevalent at P8. Scale bars, 100 μm. **b** Increment of enlarged type II cells and senescence-associated β-galactosidase (SA-β-gal) positive cells during in vitro culture. Each point corresponds to the mean and SD for at least three independent experiments at each passage. **c** Representative images of SA-β-gal staining during in vitro culture. SA-β-gal-positive enlarged hBM-MSCs were observed after P5 (indicated with white arrows) and increased in prevalence at P7 and P9. Mean and SD are shown. Scale bars, 100 μm. **d** Growth kinetics of hBM-MSCs during passaging. Data from three donors are presented and scored as population doubling time (PDT) plotted against passage. **e** Evaluation of mesodermal differentiation potential of hBM-MSCs at P2, P5 and P8 in terms of adipogenesis (Oil red O), chondrogenesis (Alcian blue) and osteogenesis (silver nitrate). Scale bars, 100 μm. **f** Changes in metaphase cell count from three donors. The number of available metaphases was decreased after P5. **g** Single-nucleotide microarray analysis of copy number alterations (CNAs) at P2, P4, P6 and P8 from three donors. Red circle indicates a small CNA at chromosome 7 that was first detected at P6 and was maintained to P8 in MSC3. See also Supplementary Fig. 1S

Most of the hBM-MSCs were homogeneous fibroblast-like type I cells in early passage (99.5 ± 0.5% at P2, 98.6 ± 0.1% at P3). Enlarged and flat-shaped epithelioid type II cells containing intracellular debris and granules were gradually increased and replaced by type I cells after in vitro culture (Fig. 1a, b). Staining for senescence-associated β-galactosidase (SA-β-gal) demonstrated that the increment of type II cells at late passage was accompanied with cellular senescence (Fig. 1c). SA-β-gal-positive cells were 0.9 ± 0.4% at P2, which was maintained at below 3% until P5. They were increased after P5, and continued to increase at P6 (19.8 ± 4.1%) and P7 (50.2 ± 6.9%). A P9, most cells stained for SA-β-gal. Average population doubling time (PDT) at less than P3 was 34.5 ± 5.9 h, which was gradually increased after P4 and P5 (46.1 ± 8.4 h) and markedly increased after P6 (63.4 ± 9.4 h). The PDT was 190.8 ± 60.5 h at P8 and no proliferation was evident at P9 (Fig. 1d).

hBM-MSCs at P2 presented typical immunophenotype of MSC, which were maintained after passaging (Supplementary Figure 1SA). hBM-MSCs at P2 and P5 demonstrated the capability of mesodermal differentiation into adipogenic, chondrogenic and osteogenic cells. However, cells in P8 revealed restricted differentiation because of reduced proliferation activity (Fig. 1e). hBM-MSCs expressed the genes representing their stemness nature in similar level, and did not express *HLA-DR* throughout all passages (Supplementary Figure 1SB). The mRNA levels of genes representing stemness and differentiation were similar in all passages (Supplementary Figure 1SC).

### Genetic characteristics of hBM-MSCs during in vitro culture

Chromosome analysis showed that all metaphases were normal across passages (Supplementary Figure 1SD). We obtained enough metaphase cells (at least 15) until P5, but failed to do so after P6 (Fig. 1f). We also performed single-nucleotide polymorphism (SNP) array analysis to investigate the genome stability at the genome-wide level. SNP array revealed 45 copy number alterations (CNAs; 25 gains and 20 losses). Hierarchical cluster analysis revealed similar patterns according to donor, with no difference based on passage. SNP array identified CNA acquired specifically during culture in MSC3. The gain of 369,948 bp at 7p22.1-p21.3 was observed from P6, and was possibly acquired during passaging (Fig. 1g). The genes in this region include *C1GALT1*, *COL28A1* and *MIOS*. However, changes in the copy number of these genes were not associated with an increment in gene expression.

### Accumulation of DNA damage and decline in telomerase activity during in vitro culture

**Fig. 2:**
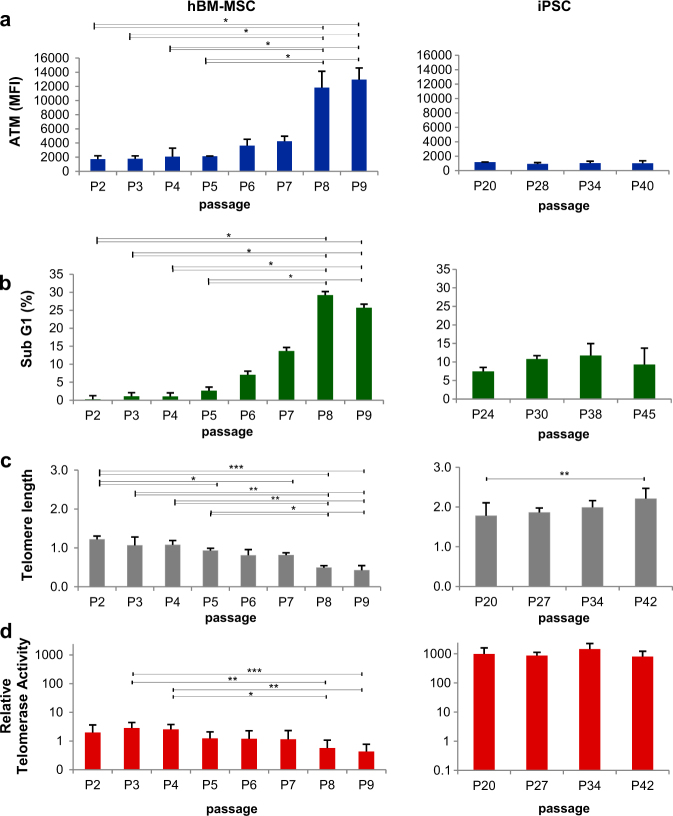
Comparison of characteristics of hBM-MSCs and iPSCs during in vitro culture **a** ATM expression was increased in hBM-MSCs (left) during passaging while the expression was maintained in iPSCs (right). **b** Sub G1 populations increased in hBM-MSCs (left) during passaging while they did not change in iPSCs (right) (see also Supplementary Fig. 2S). **c** Telomere length shortening occurred in hBM-MSCs (left) during passaging, whereas it did not in iPSCs (right). **d** Relative telomerase activities (RTA) were low in hBM-MSCs and decreased at passaged cells (left), whereas they were maintained at high level in iPSCs (right) during passaging. Results are represented as mean and SD from three independent experiments. ^*^P < 0.05, ^**^P < 0.01, ^***^P < 0.001

ATM is a protein kinase that becomes activated in response to DNA damage, particularly when the damage involves formation of DNA double-strand breaks (DSBs). Mean fluorescence intensity(MFI) value of ATM was 1707.0 ± 487.2 at P2 and was markedly increased at P6 (3616.0 ± 915.4) and at P8 (11830.5 ± 2314.4; Fig. 2a).

The proportion of S and G2M-phase cells was virtually identical, whereas the percentage of sub G1-phase cells increased during passaging (Supplementary Figure 2SA). The proportion of sub G1-phase cells was 0.2 ± 0.2% at P2 and was maintained at < 5% until P5 (4.4 ± 2.7%). Thereafter, the proportion of sub G1-phase cells increased to 8.0 ± 2.8% at P6 and 25.4 ± 4.3% at P9 (Fig. 2b). MFI of ATM was highest in G2M-phase cells followed by S, G1 and sub G1-phase cells in all passages. In contrast, ATM and cellular DNA content of iPSCs did not change during in vitro culture (Fig. 2a, b and Supplementary Figure 2SC). These data demonstrate that replication stress in hBM-MSCs leads to continuous accumulation of DNA damage and the basal level of DNA damage progressively increases in all cell cycles during passaging.

Changes in telomere length were evaluated using real-time quantitative PCR (RT-qPCR)^8^. The relative telomere to single copy gene (T/S) ratio was 1.22 ± 0.08 at P2 and decreased to 0.43 ± 0.12 at P9, giving a telomere shortening during passaging while the T/S ratio of iPSCs was 1.78 ± 0.33 until P20 and was not decreased with increased passage number (Fig. 2c). In humans, most somatic stem and progenitor cells express low levels of telomerase^9^. The basal telomerase activity from P2 to P4 was 2.4 ± 1.5 and was maintained at < 5 during subculture, while relative telomerase activity was high in iPSCs from P20 to P42 (652.4 ± 98.8). Despite low levels of telomerase activity in early passage hBM-MSCs, it was significantly phased down to 1.2 ± 1.0 (P5 to P7) and 0.5 ± 0.4 (P8 and P9) during in vitro culture(*P* = 0.0167; Fig. 2d).

### Regulatory signaling network based on altered gene expression during in vitro culture

**Fig. 3:**
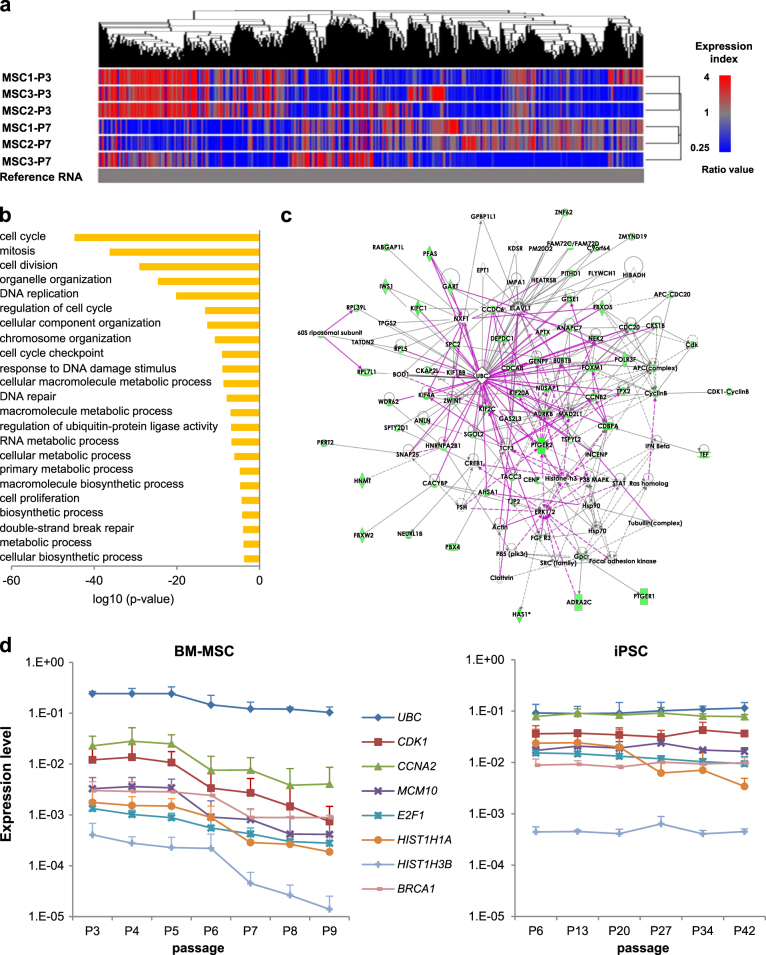
Gene expression profiles in replicative senescence of hBM-MSCs. **a** Heatmap showing the expression levels of the differentially expressed genes between P3 and P7 in hBM-MSCs ≥ 4 or ≤ 4. The dendrogram was derived by unsupervised hierarchical clustering of gene expression profiles that characterized the replicative senescence of hBM-MSCs. The algorithm grouped according to their similarity in gene expression profiles. Red spots indicate upregulated transcripts and blue spots indicate downregulated transcripts, relative to the reference RNA used. **b** General functional classification of genes downregulated more than 4-fold in late passage hBM-MSCs (P7) compared to early passage (P3). Gene Ontology (GO) analysis within the target genes of significantly altered RNAs after passaging was performed using the database for annotation, visualization and integrated discovery (DAVID) bioinformatics tool. The enriched GO biological processes were identified and listed according to their enrichment *P*-value (*P* < 0.005). The *P*-values were obtained from the DAVID 2.1 statistical function classification tool. **c** Ingenuity Pathway Analysis (IPA) network diagram illustrating annotated interactions between genes affected by in vitro culture. Network represents the merged view of the four significant subnetworks categorized by IPA function: Cell Morphology, Cellular Assembly and Organization, Cellular Function and Maintenance, Cell Cycle, Cellular Assembly and Organization, DNA Replication, Recombination, and Repair, and Cancer, Organismal Injury and Abnormalities, Reproductive System Disease. **d** Changes in *UBC*, *CDK1*, *CCNA2*, *MCM10*, *E2F1*, *HIST1H1A*, *HIST1H3B* and *BRCA1* mRNA expression of hBM-MSCs (left) during in vitro culture compared to those of iPSCs (right). Data are presented as mean and SD from three independent experiments. See also Supplementary Fig. 3S

We analyzed mRNA expression profile at P3 and P7 cells from three different donors to determine the change of mRNA expression during long-term culture of hBM-MSCs. In general, 16,095 genes revealed a 2-fold change when the expression of P7 was compared to P3 as standard. The number of genes that was two-fold downregulated (*n* = 10,820) were twice the number of upregulated genes(*n* = 5275). Among them, the number of 4-fold downregulated genes(*n* = 3582) was much higher than that of 4-fold upregulated genes (*n* = 114). Therefore, this analysis revealed that the vast majority of genes examined were significantly downregulated during passaging. Hierarchical clustering graphically revealed the different gene expression patterns between P3 and P7 (Fig. 3a).

To better understand the molecular basis of the differentially expressed genes, gene ontology(GO) and functional enrichment analysis were applied using GO Consortium terms^10^. The enriched biological processes in relation to replicative senescence of hBM-MSCs were represented in GO terms including cell cycle, cell division, organelle organization, DNA replication, cell cycle checkpoint, response to DNA damage stimulus, DNA repair, cell proliferation and DSB repair (Fig. 3b). In detail, each GO term included genes essential for the control of the cell cycle at the G1/S and G2/M phase transitions, *CDK1*, *CCNA2*; a gene involved in DNA replication, *MCM10*; a gene with a crucial role in the control of cell proliferation, *E2F1*; histone gene cluster involved in regulation of chromosome structure or function and gene expression, *HIST1H1A* and *HIST1H3B*; and the *BRCA1* gene that plays a role in maintaining genome stability and also acts as a tumor suppressor. The observed changes for selected genes were validated by RT-qPCR (all *P* < 0.05; Supplementary Figure 3SA).

The focus of this study was to elucidate candidate mechanisms of the replicative senescence in hBM-MSCs during culture. Transcriptional profiling is a valuable tool for elucidation of mechanisms underlying many biological pathways. The ingenuity pathway analysis (IPA) analysis on these gene sets indicated that the major altered Canonical Pathways were Cell Cycle Control of Chromosomal Replication, Mitotic Roles of Polo-like Kinase, ATM Signaling, Cell Cycle: G2/M DNA Damage Checkpoint Regulation and Estrogen-mediated S-phase Entry. The network indicated regulatory relationships within the genes in the data set. Seventeen distinct gene networks were observed. The main Diseases and Functions related to these network were (1) RNA Post-Transcriptional Modification, Cancer, Organismal Injury and Abnormalities of Network I; (2) Cell Cycle, Cellular Assembly and Organization of Network II; (3) DNA Replication, Recombination and Repair, Cellular Assembly and Organization and Developmental Disorder of Network IV; (4) Cell Cycle, Cellular Assembly and Organization and Cellular Function and Maintenance of Network V; (5) Cell Cycle, DNA Replication, Recombination, and Repair and Cancer of Network VI (Supplementary Figure 3SB–F). Networks III, VIII, IX, X, XI, XII, XIII and XV overlapped. It is interesting that five individual networks (X, XI, XII, XIII and XV) revealed *UBC* as a core molecule. Therefore, we tried to merge several networks and newly deduced *UBC* as a potential key regulatory molecule in replicative senescence of hBM-MSCs (Fig. 3c). In the expression microarray, *UBC* gene expression declined 0.34-fold at P7 compared to P3. This decline was confirmed by RT-qPCR as 0.50-fold (*P* = 0.009; Supplementary Figure 3SA).

To ascertain whether *UBC* is involved in the downregulation of other molecules, we performed a BisoGenet analysis including 79 cell cycle and proliferation-related genes. Most of the hub molecules identified in IPA networks were included in this interactive expanded network. The possible molecular action mechanism of *UBC* in replicative senescence of hBM-MSCs was evaluated in subsequent experiments (Supplementary Figure 3SG).

We monitored the mRNA expression of the hub molecules as well as *UBC* from P3 to P9 by RT-qPCR to determine whether the downregulation of those genes occurred gradually. The hub molecules including *CDK1*, *CCNA2*, *MCM10*, *E2F1*, *HIST1H1A*, *HIST1H3B* and *BRCA1* were sequentially decreased during passaging accompanied by the decrement of *UBC*. On the other hand, the mRNA expression of those genes was not decreased during passaging in iPSCs, which principally escapes from replicative senescence (Fig. 3d). These findings suggested that the individual hub molecules as well as *UBC* are broadly attenuated and provided indirect evidence of their cooperation in the process of replicative senescence of hBM-MSCs. The following knockdown (KD) experiment was designed to investigate the role of the *UBC* gene as a key molecule in the replicative senescence, that is involved in the control of individual hub molecules.

### *UBC* downregulation induces replicative senescence-like phenomenon

**Fig. 4:**
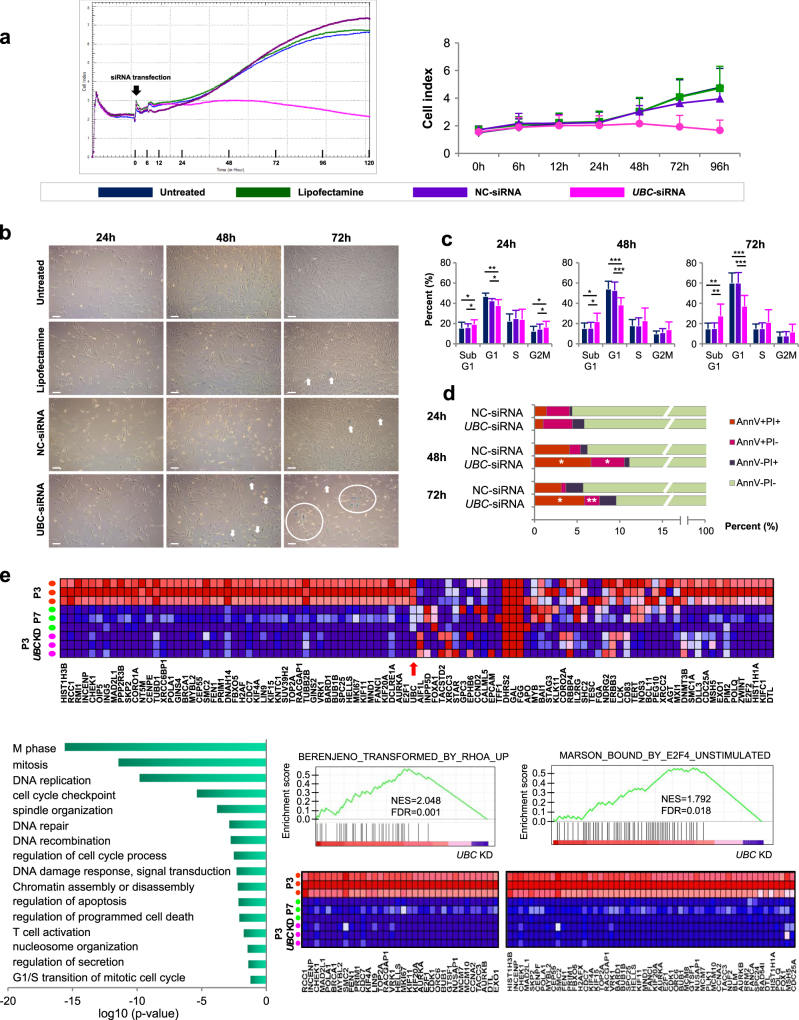
*UBC* knockdown (KD) in hBM-MSCs induces replicative senescence-like phenomenon **a** Dynamic real-time monitoring of hBM-MSC proliferation after *UBC* KD (pink line) using the xCELLigence assay compared to untreated (blue), Lipofectamine treated (green) and negative control (NC)-siRNA transfected (purple) hBM-MSCs for 120 h following treatment. Representative data are from MSC3 by three technical replicates (left) and the mean and SD from the three donors (right) (see also Supplementary Movie). **b** Expression of senescence-associated β-galactosidase (SA-β-gal) at 24, 48 and 72 h after *UBC* KD compared to untreated, Lipofectamine treated and NC-siRNA transfected hBM-MSCs. Low density cells with lower confluency with increased SA-β-gal-positivity were observed at 72 h after *UBC* KD. Scale bars, 100 μm. **c** Cell cycle changes of sub G1, G1, S and G2/M phases after *UBC* KD compared to untreated and NC-transfection. ^*^P < 0.05, ^**^P < 0.01, ^***^P < 0.001. **d** Apoptosis analysis after *UBC* KD using Annexin V/propidium iodide staining. ^*^P < 0.05, ^**^P < 0.01 compared to NC-siRNA transfected group. **e** Gene Set Enrichment Analysis (GSEA) shows similar gene expression patterns in hBM-MSCs at P7 and *UBC* KD cells at P3. Heat Map of the top 50 features for each phenotype in hBM-MSCs at P7 and P3 compared to *UBC* KD (upper). Gene ontology (GO) analysis within the target genes of significantly altered RNAs in *UBC* KD hBM-MSCs (lower left). GSEA enrichment plots and corresponding heat map images in hBM-MSCs at P3 versus P7 and *UBC* KD (lower right). NES, normalized enrichment score. FDR, false discovery rate

**Fig. 5:**
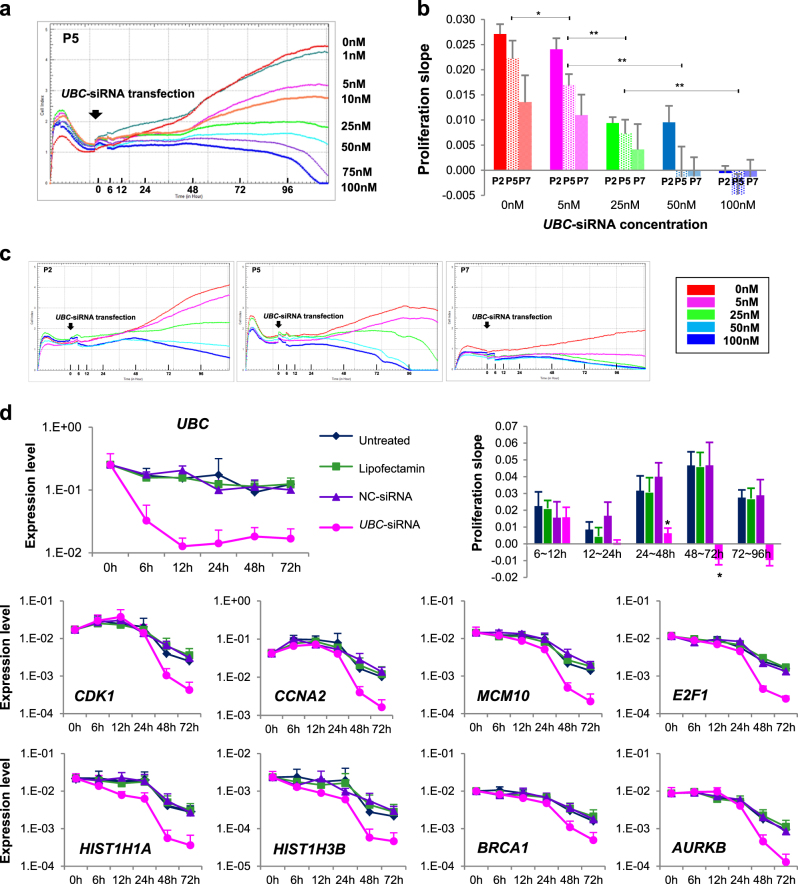
Molecular mechanism of replicative senescence induced by *UBC* downregulation **a** The degree of hBM-MSC proliferation was dependent on the concentration of the *UBC* gene expression. *UBC*-siRNA concentration ranged from 0 to 100 nM. Representative data are from MSC1 by three technical replicates. **b**, **c** Effect of the *UBC* knockdown (KD) on cell proliferation rate of hBM-MSCs at different passages (P2, P5 and P7). Cells were transfected with 0, 5, 25, 50 and 100 nM siRNA. Cell proliferation was calculated as the slope of the growth curve. Results are presented as mean ± SD from three independent experiments. ^*^P < 0.05, ^**^P < 0.01, ^***^P < 0.001 **b**. Representative data from MSC1 by three technical replicates **c** (see also Fig. 4S). **d** Observed expression changes in *UBC*, *CDK1*, *CCNA2*, *MCM10*, *E2F1*, *HIST1H1A*, *HIST1H3B*, *BRCA1* and *AURKB* genes and corresponding cell proliferation rate as slope with 100 nM *UBC*-siRNA transfected (pink line) compared to untreated (blue), lipofectamin (green) and negative control (NC)-siRNA transfected (purple) hBM-MSCs. Each point corresponds to the mean and SD for three independent experiments. *P < 0.05 versus NC-siRNA transfected group

**Fig. 6:**
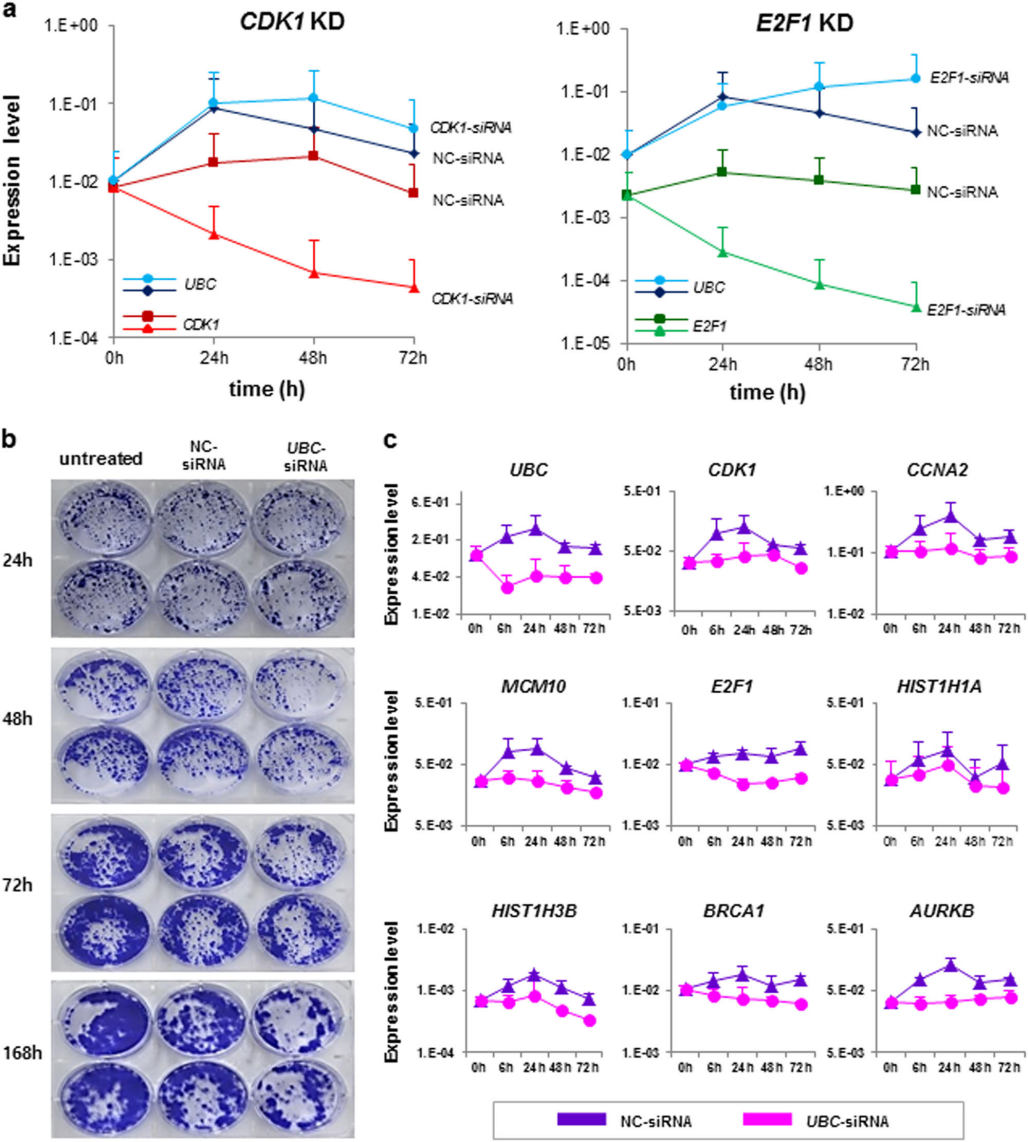
Validation of the pivotal role of the *UBC* gene in replicative senescence **a** Knockdown (KD) of *CDK1* and *E2F1* in hBM-MSCs did not influence on *UBC* gene expression. Each point corresponds to the mean and SD for three independent experiments. **b** Representative images of crystal violet-stained colonies in untreated, negative control (NC)-siRNA and *UBC*-siRNA transfected iPSCs. **c** Expression changes in *UBC*, *CDK1*, *CCNA2*, *MCM10*, *E2F1*, *HIST1H1A*, *HIST1H3B*, *BRCA1* and *AURKB* genes in *UBC*-siRNA transfected (pink line) compared to NC-siRNA transfected (purple) iPSCs. Error bars in all panels represent mean and SD from three independent experiments

On the basis of the previous results, we performed *UBC* KD experiments to identify whether the impaired proliferation of hBM-MSC is induced by *UBC* decrement. When hBM-MSCs were transfected with 100 nM *UBC*-siRNA and cultured for 96 h, cell proliferation was inhibited compared to untreated, Lipofectamine treated and negative control(NC)-siRNA transfected cells. Mitosis occurred continuously in untreated, Lipofectamin treated and NC-transfected hBM-MSCs until 96 h with high confluency, while *UBC* KD cells stopped dividing after 30 h (Supplementary Movie). Cell index was inhibited by 42.2% in *UBC* KD cells compared with NC-transfected cells at 96 h after transfection (Fig. 4a). As confirmed by light optical microscopy, NC-transfected hBM-MSCs reached about 60% confluence after 72 h, compared to 25% at most in *UBC* KD cells even though they were seeded at the same density. SA-β-gal-positive senescent cells were accumulated as 4.7 ± 1.9%, 7.8 ± 1.9% and 13.7 ± 5.1% at 24, 48 and 72 h after *UBC* KD, respectively, while they were not increased in NC-transfected cells (2.6 ± 1.9%, 2.6 ± 1.3% and 0.8 ± 0.2% after 24, 48 and 72 h, respectively)(Fig. 4b). Additionally, sub G1-phase cells were gradually increased in *UBC* KD cells (19.4 ± 4.3%, 22.2 ± 8.0% and 27.8 ± 11.4% at 24, 48 and 72 h, respectively), whereas they were similar in NC-transfected cells (16.2 ± 3.5%, 15.6 ± 5.6% and 22.2 ± 8.0% at the same respective times; Fig. 4c). Annexin V positive cell fractions with and without propidium iodide (PI) uptake were higher in *UBC* KD cells compared to NC-transfected cells at 48 and 72 h (Fig. 4d). Furthermore, live cell images also approved that many hBM-MSCs were detached from the culture plates after *UBC* KD, suggesting an apoptotic cell death (Supplementary Movie). Gene expression microarray was performed using RNA samples obtained from hBM-MSCs at 48 h after *UBC* KD. GO analysis identified significantly downregulation in genes associated with M phase, mitosis, DNA replication, cell cycle checkpoint, spindle organization, DNA repair and DNA recombination in *UBC* KD hBM-MSCs. We used Gene set enrichment analysis (GSEA) to determine whether gene expression changes in *UBC* KD cells resembled those in late passage hBM-MSCs(P7). It showed similar gene expression patterns in *UBC* KD cells and late passage hBM-MSCs (Fig. 4e). To evaluate whether the impaired proliferation occurs in a dose-dependent manner, hBM-MSCs at P5 were transfected with serial concentrations of *UBC*-siRNA and their proliferation was assessed using a real-time cell monitoring system. Growth curves indicated that the impaired proliferation of hBM-MSCs occurred in a *UBC* dose-dependent manner (Fig. 5a, b). Next, hBM-MSCs at P2, P5 and P7 were transfected with 0, 5, 25, 50 and 100 nM *UBC*-siRNA and proliferation was compared according to passage and dose simultaneously. Remarkably, the cell index lined up perfectly in the order of *UBC*-siRNA concentration. Growth curves revealed the highest proliferation activity at P2 followed by P5 and P7 sequentially in hBM-MSCs from all three donors. The average slope of growth curves during 96 h showed that the higher slope was associated with an earlier passage and KD with lower *UBC*-siRNA concentration, and vice versa (Fig. 5c). There was an inverse correlation between proliferation activity and *UBC*-siRNA concentration (Supplementary Figure 4S). These results indicated that hBM-MSCs with lower *UBC* expression due to late passage and/or KD with a high concentration of *UBC*-siRNA proliferate more poorly compared to those with higher *UBC* expression. We analyzed proliferation activity and measured mRNA levels of the hub molecules at 6, 12, 24, 48 and 72 h after *UBC* KD. *UBC* expression dropped promptly to 13% of its initial level at 6 h after KD, reached the lowest value (5%) at 12 h and remained at that level till 96 h. The expression of *CDK1*, *CCNA2*, *MCM10*, *E2F1*, *HIST1H1A*, *HIST1H3B*, *BRCA1* and *AURKB* genes started to decrease at 24 h after *UBC* KD, were markedly reduced at 48 h and remained reduced until 72 h (Fig. 5d). To validate the order of *UBC* in regulatory signal network, we knockdowned *CDK1* and *E2F1* in hBM-MSCs and found that *UBC* expression was not changed after KD of hub molecules (Fig. 6a). In addition, we performed *UBC* KD in iPSCs and observed proliferation impairment through crystal violet staining assay (Fig. 6b). The expression of hub molecules was also downregulated after *UBC* KD in iPSCs (Fig. 6c). These results imply that *UBC* plays a pivotal role in the impaired proliferation activity of hBM-MSCs and works in higher-order through regulating the genes of hub molecules in signaling networks.

### *UBC* transfection increased proliferation of hBM-MSCs

**Fig. 7:**
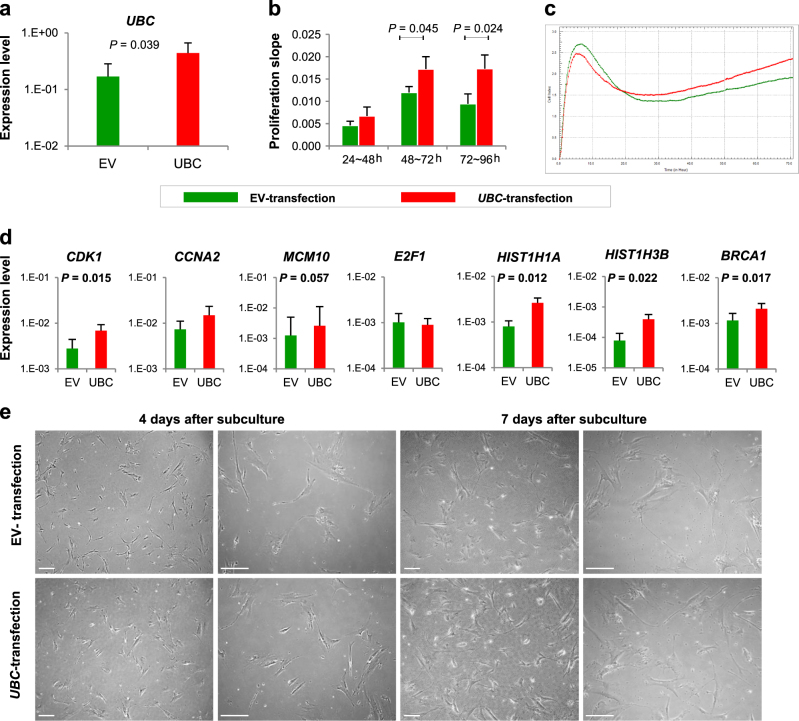
Improvement of proliferation activity by *UBC* overexpression in hBM-MSCs **a** *UBC* mRNA level after *UBC*-transfected hBM-MSCs compared to empty vector (EV)-transfected cells. **b**, **c** Comparison of cell proliferation activity by the slope of the growth curve **b** and representative image via real-time monitoring **c**. Data are presented as mean and SD; at least three independent experiments were performed. **d** qRT-PCR to analyze the mRNA level of hub molecules (*CDK1*, *CCNA2*, *MCM10*, *E2F1*, *HIST1H1A*, *HIST1H3B* and *BRCA1*) in *UBC*-transfected hBM-MSCs compared to those in EV-transfected cells. **e** Morphology and proliferation of *UBC*- and EV-transfected hBM-MSCs. The images were obtained 4 days and 7 days after subculture. Scale bars, 100 μm

As *UBC* appears to play a pivotal role in replicative senescence, we then have transfected *UBC* in hBM-MSCs at P5 and P6. The *UBC* gene expression was higher in *UBC*-transfected hBM-MSCs than in empty vector(EV)-transfected cells (*P* = 0.039; Fig. 7a). The proliferation was assessed using a real-time cell monitoring system. The average slope of growth curves during 96 h showed higher slope in *UBC*-transfected cells (Fig. 7b). Growth curves indicated that *UBC*-transfected hBM-MSCs caught up the proliferation activity of the EV-transfected cells(Fig. 7c). The adherent cell number increased with the culture time, which was higher in the *UBC*-transfected cells compared to EV-transfected cells. We measured the mRNA expression of the hub molecules by RT-qPCR and found that *CDK1*, *E2F1*, *HIST1H1A*, *HIST1H3B* and *BRCA1* were significantly increased in *UBC*-transfected cells compared to EV-transfected cells (Fig. 7d). The *UBC*-transfected hBM-MSCs revealed fibroblast-like morphology until 7 days after subculture (Fig. 7e). These results suggest that *UBC* overexpression improves proliferation activity in hBM-MSCs.

## Discussion

In this study, we induced replicative senescence in hBM-MSCs and observed the changes of combined biological and genetic characteristics during in vitro culture. Impaired proliferation, one of the principal characteristics of senescence, was coupled with gain of SA-β-gal-positive and large-sized type II MSCs as expected. hBM-MSC barely maintained the general characteristics, such as surface markers and multipotency, but the representative capacities clearly weakened during long-term in vitro culture.

Genome stability of stem cells is essential for stemness and pleuripotency. Genome instability accumulates during in vitro culture and initiates cellular senescence^11^. Replicative senescence in MSCs is intimately associated with the development of aneuploidy, which is associated with a low proliferative capacity^12^. The genetic alterations of hBM-MSCs were more frequently observed in myelodysplastic syndrome and acute myeloid leukemia patients, which revealed increased senescence and possible functional impairment compared with normal control^13^. Chromosome analysis was hindered by difficulty in obtaining an adequate number of metaphase cells, which provides further proof of impaired proliferation occurring after P5. Therefore, SNP array analyses were done presently and identified genome copy number alterations in late passage from one donor. Results from this and previous studies support the view that MSCs become senescent with or without genomic alterations^6,14^. DNA damage arising during DNA replication and recombination leads to defects in tissue maintenance and immediate cell death with proliferation arrest^15,16^. Continued accumulation of DNA damage in all cell cycles analyzed by ATM measurement and lowering telomerase activity were representative characteristic of replicative senescence. Telomere length was also shortened during passaging. On the contrary, iPSCs maintained higher telomerase activity during in vitro culture and their telomere length was not decreased. Interestingly, the percentage of sub G1-phase cells was increased during passaging. This observation suggested that both senescence and apoptosis occurred during in vitro culture^17^.

In parallel, we scrutinized gene expression changes at early and late passage using integrative signal network analyses to explore the molecular mechanism of replicative senescence in hBM-MSCs. We clearly demonstrated a senescence-associated molecular mechanism that is expressed as downregulation of proliferation machinery. Ontogenetically, it was striking to document the significantly reduced expression in senescent cells of genes involved in cell cycle, cell division, organelle organization, DNA replication, DNA packaging, cell cycle checkpoint, response to DNA damage stimulus, DNA repair, RNA metabolic process, regulation of protein, cell proliferation and DSB repair. Notably, through integrated *in silico* signal network analysis, *UBC* emerged as a pivotal player in replicative senescence of hBM-MSCs. It is central in several networks that merged into a conjoined network. Furthermore, *UBC* seemed to affect the molecules functioning at cell cycle regulation and proliferation which were decreased alongside *UBC* during passaging. *UBC* is a stress responsive gene that is fundamentally important in the responses to external threats^18^. The importance of the *UBC* gene was highlighted in the knockout mouse. Disruption of *UBC* in mice is embryonically lethal combined with reduced growth rates, premature senescence, increased apoptosis and delayed cell-cycle progression and increased susceptibility to cellular stress of mouse embryonic fibroblasts^19^.

The broad attenuation of *UBC* and other hub molecules in hBM-MSCs during subculture was averted in iPSCs that evade replicative senescence. Counteraction of senescence-associated modifications seems to play a central role in the generation of iPSCs and all signs of cellular aging seem to be removed upon reprogramming into iPSCs^20^. The maintained expression of *UBC* and hub genes during subculture guards iPSCs from replicative senescence. Changes of senescence-associated gene expression were not restricted to late senescent passages, but increased continuously during in vitro culture. This is in line with the continuous changes in biological and genetic characteristics. This finding is consistent with results from a previous study, which also indicated that, on a molecular basis, replicative senescence is a continuous process starting from the first passage onwards^21^.

To corroborate the function of *UBC* as a guide of replicative senescence, we undertook an in vitro KD study using siRNA. The real-time cell monitoring system and live cell imaging revealed the impaired proliferation distinctly beginning 24 h after transfection. Cell index was inhibited by 53.0% and 42.2% in *UBC* KD hBM-MSCs compared with NC-transfected cells at 72 and 96 h after transfection, respectively. This impaired proliferation due to induced senescence was verified by lower confluency and higher SA-β-gal-positive cell fraction. We also observed that many *UBC* KD cells were detached from the culture plates, suggesting apoptotic cell death which was also confirmed by the increased Annexin V positive sub G1-phase cells. This and previous observation raises the possibility that senescence and apoptosis pathways simultaneously engaged in certain processes or stress responses such as repetitive subculture^17^.

The decreased proliferation occurred in dose-dependent manner in hBM-MSCs transfected by higher concentration of *UBC*-siRNA demonstrated poorer cell index. This indicated that the gradual impairment of proliferation activity during in vitro culture could be attributed to decreased *UBC* expression. Gene expression changes in *UBC* KD cells were similar to late passage hBM-MSCs. Similar phenomenon was observed in *UBC* KD iPSCs. We also observed a knock-on effect of *UBC* KD on associated hub genes. The activity of these genes began to decrease at 12 h after the lowest attained *UBC* level. Inversely, KD of *CDK1* and *E2F1* did not influence the *UBC* gene expression. It is noteworthy that hBM-MSCs revealed improved proliferation activity after *UBC* overexpression accompanied by increased expression of the hub genes. These results imply that *UBC* works in higher-order through regulating the series of genes controlling cell cycle and proliferation, and induces replicative senescence in hBM-MSCs.

Senescence is a multi-step evolving process and there are several types of senescence whose mechanism and biological roles are not fully understood^22^. Each senescent phenotype has specific features and it is noteworthy to find specific molecular signature for each of them. Replicative senescence is considered as chronic senescence by prolonged periods of cellular stress^23^. Because the senescent cells are heterogeneous, we cannot explain the entire replicative senescence through *UBC* decrement alone. Further study is needed to refine our understanding of the relationship between replicative senescence and *UBC* decrement.

In summary, replicative senescence in hBM-MSCs is characterized as deterioration in representative cell characteristics, accumulated DNA damage, and decreased telomere length and telomerase activity with or without genomic abnormalities. Replicative senescence results from the continued downregulated expression of a series of genes, with *UBC* decrement as a pivot in the molecular mechanism of this regulation.

## Materials and methods

### Evaluation of characteristics of human bone marrow-mesenchymal stromal cells (hBM-MSCs) during repetitive subculture

#### hBM-MSC culture

This study was approved by the Institutional Review Board, Seoul St. Mary’s Hospital (IRB approval number KC12CNSI0078). Three preparations of hBM-MSCs (donor age: 24, 27 and 22 year) at passage one (P1) were purchased from Texas A&M Health Science Center, College of Medicine, Institute for Regenerative Medicine (Temple, TX, USA). To recover viable cells, a frozen vial of the hBM-MSCs was rapidly thawed, plated in a 150 × 25 mm culture dish and cultured using alpha-modified minimum essential medium (α-MEM; Gibco, Grand Island, NY, USA) containing 16.5% fetal bovine serum (FBS; Gibco), 1% glutamax-I (Gibco) and 1% penicillin-streptomycin (Gibco) at 37 °C and 5% CO_2_^24^. Next day, the adherent cells were detached using 0.25% trypsin-EDTA (Gibco) and cells were re-plated at a density of 300 cells/cm^2^. At this time, cells were considered P2 and medium was changed every 3–4 days. Cultured hBM-MSCs were harvested at 70–80% confluency by treatment with 0.25% trypsin-EDTA (Gibco). The number of harvested cells was counted using a C-Chip hemocytometer (SystemBükerTürk; Incyto, Cheonan, Korea) and cells were re-plated at a density of 300 cells/cm^2^. These processes continued until hBM-MSCs ceased proliferating. The proliferative activity of hBM-MSCs was expressed as population doubling time (PDT)^6^.

#### Immunophenotyping

hBM-MSC immunophenotypes were evaluated during long-term culture^25^. Harvested cells were labeled using mouse anti-human monoclonal antibodies: phycoerythrin (PE)-conjugated CD105, CD90, CD73, CD34, CD45, CD11b and CD79a, and fluorescein isothiocyanate (FITC)-conjugated HLA-DR (BD Biosciences, San Jose, CA, USA). As a control, isotype PE-conjugated IgG1 and FITC-conjugated IgG2a (BD Biosciences) were used. The cell suspension containing 1 × 10^6^ cells was incubated with monoclonal antibodies for 15 min at room temperature in the dark and fixed with BD Cytofix (BD Biosciences). The samples were analyzed on FACSCalibur cytometer (BD Biosciences) and the resulting data were processed using CellQuest Proversion 6.0 software (BD Biosciences).

#### MSC-specific stemness and differentiation gene expression

RNA was extracted using the RNeasy Mini Kit (Qiagen, Hilden, Germany) according to the manufacturer’s instructions. cDNA was synthesized with 2 μg of RNA using Transcriptor First strand cDNA synthesis kit (Roche, Mannheim, Germany). In order to confirm hBM-MSCs stemness nature, reverse transcription-polymerase chain reaction (RT-PCR) was performed with 8 specific primers including octamer-binding transcription factor 4 (*OCT-4*), homeobox transcription factor Nanog (*NANOG*), SRY-related HMG-box 2 (*SOX2*), GATA-binding factor 4 (*GATA-4*), stem cell factor (*SCF*), telomerase reverse transcriptase (*TERT*), *HLA-ABC* and *HLA-DR*. The primers sequences are presented in Supplementary Table 1S. Glyceraldehyde-3-phosphate dehydrogenase (*GAPDH*) was the internal control. PCR conditions were 5 min at 96 °C, 35 cycles of 30 s at 96 °C, 30 s at 58 °C, 30 s at 72 °C and 5 min at 72 °C. PCR products were electrophoresed on 2% agarose gels with GelRed Nucleic Acid Stain (Biotium, Hayward, CA, USA) and bands were observed under ultraviolet light.

In addition, real-time quantitative PCR (RT-qPCR) was performed with primers of the stemness and differentiation genes including adiponectin (*ADPQR*), SRY-related HMG-box 9 (*SOX9*), collagen type I alpha 1 chain (*COL1A1*) and osteocalcin (*BGLAP*). RT-qPCR was performed using PowerUp SYBR green master mix (Applied Biosystems, Foster City, CA, USA) according to the manufacturer’s instructions. PCR reactions were run on a ViiA 7 Real-Time PCR system (Applied Biosystems) under standard conditions. After PCR, a dissociation curve (melting curve) was constructed in the range of 60 °C to 95 °C. The gene expression was normalized with a reference gene, *GAPDH*. All experiments were repeated three times to test reproducibility.

#### In vitro mesodermal differentiation

Maintenance of mesodermal differentiation potential of hBM-MSCs was examined during long-term culture against P2, P5 and P8 cells. Cells (1 × 10^5^) were dispensed in wells of in 6-well culture dishes (Nunc, Shanghai, China)^26^. After hBM-MSCs reached 70–80% confluency, the medium was removed and replaced with an adipogenic, osteogenic and chondrogenic differentiation medium. Cells were incubated at 37 °C for 3 weeks with biweekly change of the medium.

Adipogenic differentiation was induced using adipogenesis differentiation basal medium (Gibco) containing 1 μM dexamethasone, 200 μM indomethacin and 0.5 mM isobutylmethylxanthine. After 3 weeks, cells were fixed with 4% paraformaldehyde (PFA) solution and stained using Oil Red-O (Sigma-Aldrich, St. Louis, MO, USA) to reveal intracellular lipid accumulation as a means of assessing adipogenic differentiation potential.

For chondrogenic differentiation, 2.5 × 10^5^ cells were centrifuged at 150 × g for 5 min in a 15 mL conical tube (BD Biosciences), then cultured with chondrogenesis differentiation basal medium (Gibco) supplemented with 10 ng/ml transforming growth factor-β1 and 50 nM ascorbic acid-2-phosphate. After 3 weeks, cell pellets were fixed with 4% PFA and embedded in paraffin. Sections were stained with 1% Alcian blue (Sigma-Aldrich). Nuclei were stained with Nuclear Fast Red solution (Sigma-Aldrich).

Osteogenic differentiation was induced by a specific medium (Gibco) supplemented with 100 nM dexamethasone, 50 μM ascorbic acid-2-phosphate and 10 ng/mL recombinant human bone morphogenetic protein. After 3 weeks, calcium deposits in the cells were stained by the von Kossa method, and observed under the microscope.

#### Senescence-associated β-galactosidase (SA-β-GAL) activity assay

The most widely used biomarker for senescent and aging cells is SA-β-gal, which is defined as β-galactosidase activity detectable at pH 6.0 in senescent cells^27^. hBM-MSCs were stained using SA-β-gal staining kit (Cell Signaling Technology, Boston, MA, USA). Cells were seeded (300 cells/cm^2^) in wells of 6-well culture dishes and cultured. After 4 days, cells were stained at pH 6.0. Senescent cells were identified as blue-stained cells under inverted microscopy examination. The percentage of senescent cells was obtained by counting the number of blue-stained cells in at least 10 random fields.

### Evaluation of genetic stability

#### Conventional chromosome analysis

Genetic stability was initially evaluated using conventional chromosome analysis. Cultured hBM-MSCs were treated with 10 μg/μL Colcemid (PAA Laboratories GmbH, Pasching, Austria) for 4 h. The cells were harvested and treated with hypotonic KCl (0.075 M) solution and fixed with Carnoy’s solution^13^. G-banding was performed and karyotype was described according to the International System for Human Cytogenetic Nomenclature recommendations (2013)^28^.

#### Combined array comparative hybridization plus single-nucleotide polymorphism microarray (CGH + SNP microarray) analysis

Genomic DNA was extracted using a Wizard Genomic DNA Purification Kit (Promega, Madison, WI, USA) from hBM-MSCs at P2, P4, P6 and P8. DNA concentration and purity was checked using a ND-1000 spectrophotometer (Nanodrop Technologies, Wilmington, DE, USA). DNA quality was confirmed by 2% agarose gel electrophoresis with reference DNA (Agilent Technologies, Santa Clara, CA, USA) to rule out degradation. To determine the copy number alterations (CNAs) during passaging of hBM-MSCs, we used a SurePrint G3 Human CGH + SNP Microarray 4 × 180 K kit (Agilent Technologies). Agilent’s male and female genomic DNA (European normal individual) was used as reference DNA. DNA 1.5 μg was digested using AluI and RsaI at 37 °C for 2 h. Each digested sample was labeled using Cy5-dUTP for hBM-MSCs, Cy3-dUTP for reference DNAs. After clean-up with SureTag DNA Labeling Kit Purification Columns (Agilent Technologies), hybridization was performed on a microarray slide that was rotated at 65 °C for 24 h. The microarray slide was scanned and the raw data extracted using Agilent Feature Extraction software V10.7.3.1. The raw data was analyzed using Genomic Workbench 7.0.4.0 software (Agilent Technologies) under the default settings with a slight modification; ADM-2 algorithm with a threshold of 6, minimum absolute average log2 ratio in minimum number for Amplification or deletion of 3 consecutive probes. Loss of heterozygosity (LOH) was considered using the LOH algorithm at the default threshold of 6.0. Probe mapping was conducted according to its genomic location in the UCSC genome browser (Human NCBI37/hg19).

#### Flow cytometry assessment of DNA damage and cell cycle

DNA damage was assessed by the phosphorylation of the histone variant H2AX (γH2AX) using FlowCellect^TM^ Cell Cycle Checkpoint Ataxia telangiectasia mutated (ATM) DNA Damage Kit (Millipore, Temecula, CA, USA)^29^. Briefly, a test sample of 1 × 10^6^ cells was fixed, permeabilized and labeled with Alex Fluor 488-conjugated anti-phospho-ATM on ice for 30 min in the dark. DNA was stained with PI at room temperature in the dark for 30 min. At least 20,000 events were analyzed using a FACS Calibur cytometer (BD Biosciences) and ATM enzyme activity and cell distribution in each phase of the cell cycle including sub G1-peak of apoptotic cells were analyzed using CellQuest Pro version 6.0 software (BD Biosciences) as described previously^30^.

#### Flow cytometry assessment for detection of apoptosis

Cell apoptosis was evaluated using Annexin V-fluorescein isothiocynate (FITC) apoptosis detection kit (Abcam, Cambridge, UK) according to the manufacturer’s instructions. The cell suspension of 1 × 10^5^ cells was stained FITC-conjugated Annexin V and PI in the dark for 15 min at room temperature. Then, the stained cells were analyzed by FACS Calibur cytometer and CellQuest Pro version 6.0 software (BD Biosciences).

#### Telomerase activity assay

Telomerase activity was measured using the telomerase rapid amplification (TRAP) assay using the TeloTAGGG Telomerase PCR ELISA^PLUS^ kit (Roche). 2 × 10^5^ cells were lysed and then 1.5 μg of supernatant was used for the PCR reaction. For PCR reaction, each supernatant was divided into two aliquots. One aliquot of the supernatant was mixed with the TeloTAGGG Telomerase reaction mixture containing the internal standard. The other aliquot of the supernatant was incubated at 85 °C for 10 min to inactivate the telomerase protein to produce negative control prior to mixing with the TeloTAGGG Telomerase reaction mixture containing internal standard. The entire reaction mixture was amplified under the following conditions: 30 min incubation at 25 °C for primer elongation, 30 cycles of denaturation for 30 s at 94 °C, annealing for 30 s at 50 °C and polymerization for 1.5 min at 72 °C with a final step of 10 min at 72 °C. The PCR products were denatured and hybridized to a digoxigenin-labeled probe and detected by ELISA. The level of telomerase activity in a sample was determined by comparing the signal from the sample to the signal obtained using a known amount of a control (relative telomerase activity; RTA, percentage of telomerase activity in comparison with iPSCs). Induced pluripotent stem cells (iPSCs) which were kindly donated by Professor Jangwhan Kim, Korea Research Institute of Bioscience and Biotechnology were used as control.

#### Telomere length analysis

We evaluated the change of telomere length during passaging using real-time quantitative RT-PCR (RT-qPCR)^8^. Telomere-specific primers (forward: 5′-CGGTTTGTTTGGGTTTGGGTTTGGGTTTGG GTTTGGGTT-3′; reverse:5′-GGCTTGCCTTACCCTTACCCTTACCCTTACCCTTACCCT-3′) and the 36b4 primers (forward: 5′-CAGCAAGTGGGAAGGTGTAATCC-3′; reverse: 5′-CCCATTCTATCATCA ACGGGTACAA-3′ were used. All PCRs were performed on the Rotor-Gene Q real-time instrument (QIAGEN). Thermal cycling profile for both amplicons began with at 95 °C incubation for 10 min. For telomere PCR, there followed 25 cycles of 95 °C for 15 s, 58 °C for 1 min. For 36B4 PCR, there followed 30 cycles of 95 °C for 15 s, 58 °C for 1 min. Rotor-Gene Q software 2.0.2 was then used to determine the telomere-to-Single copy gene (T/S) ratio, which is demonstrated to be proportional to the average telomere length in a cell.

#### Gene expression analysis by microarray

To compare the gene expression profile of hBM-MSCs at early (P3) and late passage (P7), we performed one-color microarray-based gene expression analysis. RNA was quantified using the aformentioned Nanodrop spectrometer and the quality was confirmed using a model 2100 Bioanalyzer (Agilent Technologies). Gene expression was examined using SurePrint G3 Hmn GE 8 × 60 K V2 Microarray Kit (Agilent Technologies) according to the manufacturer’s instructions. Briefly, 200 ng of total RNA was biotinylated and purified using Low Input Quick Amp Labeling Kit (Agilent Technologies) and synthesized to double-stranded cRNA. Subsequently, 600 ng of cRNA was hybridized according to the manufacturer’s instructions. GeneChip arrays were scanned in a high-resolution microarray scanner (Agilent Technologies). The average fluorescence intensity for each spot was calculated and local background was subtracted with the Agilent Feature Extraction software package. All data normalization and selection of upregulated and downregulated genes was performed using GeneSpring GX 7.3 (Agilent Technologies). The averages of normalized ratio were calculated by dividing the average of the test channel signal intensity by the control channel signal intensity. Functional annotation of genes was performed according to the Gene Ontology Consortium (http://www.geneontology.org/index.shtml) by GeneSpringGX 7.3. Gene classification was based on searches done by GeneCards (http://www.genecards.org/) and DAVID (http://david.abcc.ncifcrf.gov/).

#### Integration of gene network analysis

The “Core Analysis” function was used to interpret the data in the context of biological processes, pathways and networks. Gene network analysis was performed for representation of relationships among biomolecules using the Ingenuity® Pathway Analysis (IPA; Qiagen) and BisoGenet. IPA is a functional analysis tool that can identify the most relevant signaling and metabolic pathways, molecular networks and biological functions for a list of genes^31^. Genes that displayed a 4-fold upregulation or 8-fold downregulation in P7 compared to P3 (common up- or downregulated genes) were uploaded to IPA for signaling network and functional analyses. The network analysis showed interrelatedness between these gene sets. The association of the gene sets with the canonical pathways indicated possible effects on the well-defined biological pathway of the IPA platform, based on the most updated knowledge base. Upregulated and downregulated identifiers identified the relationship between gene expression alterations and related changes in biofunctions under the subcategories of Molecular and Cellular Functions, Physiological System Development and Function, and Disease and Disorders. The network was ranked according to a score representing the probability that each isolated network of genes could be achieved by chance alone. A score >3 was considered significant; with this score, there is a 1 in 1000 chance that the focus genes in the network were isolated because of random chance (scores of 3 have a 99.9% confidence level of not being generated by random chance alone).

BisoGenet is a client-server based multitier application designed as Cytoscape plugin that is a software platform for visualization and analysis of biomolecular relationships^32^. Genes in cellular proliferation signal network were selected and build a new integrated network by BisoGenet software, incorporating coding relations.

We chose to use Gene Set Enrichment Analysis (GSEA) to analyze functionally related groups of genes altered by *UBC* knockdown (KD)^33^. Each of the genes was calculated for the extent of differential expression between P3, P7, or *UBC* KD P3 hBM-MSCs and reference RNAs. For GSEA, genes with more than 2-fold changes were extracted. Thereafter, we identified for the functional relevance of genes altered in the P7 and *UBC* KD hBM-MSCs compared to P3.

#### Gene expression analysis by RT-qPCR

To confirm the gene expression level, we selected eight genes (*CDK1*, *CCNA2*, *MCM10*, *E2F1*, *HIST1H1A*, *HIST1H3B*, *BRCA1* and *UBC*) that were identified as hub molecules in network analysis. The eight genes were subjected to RT-PCR analysis of hBM-MSCs from P3 to P9. RT-qPCR was carried out in triplicate using the TaqMan gene expression assay (Applied Biosystems, Foster City, CA, USA) and TaqMan gene expression master mix (Applied Biosystems) according to the manufacturer’s instructions. Probe information is described in Supplementary Table 2S. PCR reactions were run on a ViiA 7 Real-Time PCR system (Applied Biosystems) under standard conditions. *GAPDH* was selected as a reference gene for normalizing mRNA level. In addition, RT-qPCR was performed in iPSCs at P6, P13 and P20 to compare the gene expression changes during in vitro culture.

### Knockdown experiment and real-time cell monitoring

#### Small interfering RNA (siRNA) transfection

Since the gene network analysis demonstrated that the *UBC* gene might be a key molecule of cellular senescence, *UBC* gene KD was performed by siRNA transfection into hBM-MSCs and P20 iPSCs. The specific sequence for human *UBC* gene siRNA (5′-GUGACACUAUCGAGAACGUTT-3′) was designed and synthesized by GenePharma (Shanghai GenePharma, Shanghai, China). hBM-MSCs at third passage were transfected with 100 nM *UBC*-siRNA using Lipofectamine RNAiMAX (Invitrogen Life Technologies, Carlsbad, CA, USA) according to the manufacturer’s instructions. After a 6-h transfection, 2 mL of basic cell culture medium containing 1% antibiotics was replaced in each well. MSCs were cultured for an additional 3 days. Untreated, Lipofectamine RNAiMAX treated and negative control siRNA (GenePharma) (NC)-treated hBM-MSCs were used as controls. NC is not homologous with the target gene; transfection with the same approach used with *UBC* siRNA-transfection was done. Total RNA was prepared for gene expression microarray and RT-qPCR at 0, 6, 12, 24, 48 and 72 h after transfection.

Next, hBM-MSC at P5 were transfected with 1, 5, 10, 25, 50, 75, 100 nM *UBC*-siRNA and proliferation activity was analyzed using a real-time cell monitoring system. hBM-MSCs at P2, P5 and P7 were transfected with 0, 5, 25, 50 and 100 nM *UBC*-siRNA and proliferation was compared according to passage and dose simultaneously.

We performed additional KD experiments using 100 nM siRNAs against *CDK1* and *E2F1* genes to verify *UBC* worked in higher-order compared to the hub genes controlling cell cycle and proliferation. KD was performed by siRNA transfection into hBM-MSCs. The specific sequence for human *CDK1* and *E2F1* genes siRNA (5′-CCUGGUCAGUACAUGGAUUTT-3′, 5′-CCUCUUCGACUGUGACU UUTT-3′, respectively) were designed and synthesized by GenePharma (Shanghai GenePharma). Transfection was performed as described above and total RNA was extracted at 0, 24, 48 and 72 h after transfection for RT-qPCR.

#### Real-time cell assessment of cellular proliferation

The effects of the *UBC* gene KD and transfection on hBM-MSC proliferation were monitored using xCELLigence RTCA DP system (Roche), which allows for label-free, real-time monitoring of the cultured cell viability using impedance^34^. A total of 50 μL of antibiotic-free basic cell culture medium was added to an E-plate with plate bottom coated with gold electrodes. After incubation at room temperature for 15 min, the background intensity was measured. Cells (*n* = 10,000) were suspended in 150 μL of antibiotic-free basic cell culture medium and seeded in each well of the E-plate. Twenty-four hours after E-plate was installed in the xCELLigence system, *UBC* gene KD was performed combined with control treatments. The cell index was measured every 10 min, which is a relative values calculated by analyzer. Data analysis was carried out using RTCA software 2.0 supplied with the instrument. All experiments were performed in triplicate.

#### Live cell image analysis for assessment of cellular proliferation

Live imaging of the effects of *UBC* gene KD on hBM-MSC proliferation was done using a model DMI6000 B analyzer (Leica Microsystems, Wetzlar, Germany) equipped with an Adaptive Focus Control system as part of an automated/motorized inverted microscope. The atmosphere in the incubator was humidified and set to 37 °C with 5% CO_2_. Images were taken at 200-fold magnification at 15 min intervals from 6 to 96 h post-transfection, and were analyzed using LAS AF software (Leica Microsystems).

### Transfection of *UBC* gene

Lentiviral pEZ-Lv205 empty vector and pEZ-Lv205 *UBC* (GeneCopoeia, Rockville, MD) were prepared for transfection. hBM-MSCs were seeded onto 4 × 10^4^ in each well of a 6-well plate (Life Technologies) using transfection medium containing α-MEM (Gibco) medium containing 12.5% FBS (Gibco), 1% glutamax-I (Gibco) without antibiotics the day before transfection. Transductions were carried out at multiplicity of infection 7.0 in the presence of transfection medium containing 8 μg/ml polybrene (Millipore). To enhance transduction efficiency, the cells were concentrated by centrifugation at 2500 rpm for 1 h and cultured at 37 °C and 5% CO_2_. After replacement of medium on the next day, the cells were cultured for additional 48 h and monitored for green fluorescent protein (GFP) expression under fluorescence microscopy. The transfected hBM-MSCs were harvested after 72 h incubation using 0.25% trypsin-EDTA (Gibco). The cells were subcultured at 900 cells/cm^2^ density in transfection medium and harvested 5 days after subculture to analyze *UBC* gene expression. The effect of *UBC*-transfection for cell proliferation was monitored in real time using xCELLigence RTCA DP system (Roche) as described above. All experiments were performed in triplicate.

### iPSCs experiment for comparison to hBM-MSCs

Cultured iPSCs were transferred onto Matrigel-coated 6-well plates containing DMEM/GlutaMAX (Life Technologies) supplemented with 10% fetal bovine serum and a Rho kinase inhibitor, Y-27632 (10 mM prepared in 100 μl of Dulbecco’s phosphate-buffered saline) (Sigma-Aldrich). After the cells adhered, the medium was changed to chemically defined Essential 8 medium (Life Technologies). The medium was changed daily, and cells were passaged every 5–6 days using Accutase Cell Detachment Solution (STEMCELL Technologies, Vancouver, Canada)^35^.

The iPSC clones were fixed with 4% paraformaldehyde and immunostaining was performed using the following primary antibodies: Sox2, Tra-1–60, SSEA-4 and Oct4 and Alexa Fluor 594-conjugates or 488-conjugated secondary antibody (Life technologies). Alkaline phosphatase live cell staining was also performed (Life Technologies). These factors all stained positively in iPSC clones which were detected by indirect immunofluorescence microscopy (Carl Zeiss, Göttingen, Germany; Supplementary Figure S2D). Cell viability and proliferation after *UBC* KD were assessed by crystal violet staining assay. Cells were stained with crystal violet (Sigma-Aldrich) at the indicated time points.

### Statistical analysis

The means of quantitative data were compared using Student’s t-test. Spearman correlation was utilized to verify the correlation between the slope of proliferation and *UBC*-siRNA concentration. A difference was considered statistically significant when *P* < 0.05. Statistical analysis was undertaken using IBM^®^ SPSS^®^, version 24.0 (IBM Corp., Armonk, NY, USA).

### Accession numbers

The microarray data have been submitted to the National Center for Biotechnology Information (NCBI; Bethesda, MD, USA) the Gene Expression Omnibus database (GEO accession number GSE88806; https://www.ncbi.nlm.nih.gov/geo/).

## Electronic supplementary material

Supplemental Information

Supplementary Movie

## References

[1] da Silva Meirelles L, Chagastelles PC, Nardi NB (2006). Mesenchymal stem cells reside in virtually all post-natal organs and tissues. J. Cell. Sci..

[2] Wang Y (2013). Long-term cultured mesenchymal stem cells frequently develop genomic mutations but do not undergo malignant transformation. Cell Death Dis..

[3] Oh J, Lee YD, Wagers AJ (2014). Stem cell aging: mechanisms, regulators and therapeutic opportunities. Nat. Med..

[4] Sepúlveda JC (2014). Cell senescence abrogates the therapeutic potential of human mesenchymal stem cells in the lethal endotoxemia model. Stem Cells.

[5] Sensebe L, Gadelorge M, Fleury-Cappellesso S (2013). Production of mesenchymal stromal/stem cells according to good manufacturing practices: a review. Stem Cell Res. Ther..

[6] Torre ML (2015). Ex vivo expanded mesenchymal stromal cell minimal quality requirements for clinical application. Stem. Cells. Dev..

[7] Barabási AL, Oltvai ZN (2004). Network biology: understanding the cell’s functional organization. Nat. Rev. Genet..

[8] Cawthon RM (2002). Telomere measurement by quantitative PCR. Nucleic. Acids. Res..

[9] Gunes C, Rudolph KL (2013). The role of telomeres in stem cells and cancer. Cell.

[10] Ashburner M (2000). Gene ontology: tool for the unification of biology. The Gene Ontology Consortium. Nat. Genet..

[11] Capelli C (2014). Frequent occurrence of non-malignant genetic alterations in clinical grade mesenchymal stromal cells expanded for cell therapy protocols. Haematologica.

[12] Estrada JC (2013). Human mesenchymal stem cell-replicative senescence and oxidative stress are closely linked to aneuploidy. Cell Death Dis..

[13] Kim Y (2015). Genetic and epigenetic alterations of bone marrow stromal cells in myelodysplastic syndrome and acute myeloid leukemia patients. Stem Cell Res..

[14] Tarte K (2010). Clinical-grade production of human mesenchymal stromal cells: occurrence of aneuploidy without transformation. Blood..

[15] Oberle C, Blattner C (2010). Regulation of the DNA damage response to DSBs by post-translational modifications. Curr. Genomics..

[16] Sperka T, Wang J, Rudolph KL (2012). DNA damage checkpoints in stem cells, ageing and cancer. Nat. Rev. Mol. Cell. Biol..

[17] Childs BG, Baker DJ, Kirkland JL, Campisi J, van Deursen JM (2014). Senescence and apoptosis: dueling or complementary cell fates?. EMBO. Rep..

[18] McBride WH, Iwamoto KS, Syljuasen R, Pervan M, Pajonk F (2003). The role of the ubiquitin/proteasome system in cellular responses to radiation. Oncogene..

[19] Ryu KY (2007). The mouse polyubiquitin gene UbC is essential for fetal liver development, cell-cycle progression and stress tolerance. EMBO. J..

[20] Koch CM (2013). Pluripotent stem cells escape from senescence-associated DNA methylation changes. Genome Res..

[21] Wagner W (2008). Replicative senescence of mesenchymal stem cells: a continuous and organized process. PLoS. ONE..

[22] van Deursen JM (2014). The role of senescent cells in ageing. Nature..

[23] Capasso S (2015). Changes in autophagy, proteasome activity and metabolism to determine a specific signature for acute and chronic senescent mesenchymal stromal cells. Oncotarget.

[24] Gronthos, S., & Zannettino, A. C. W. *Mesenchymal Stem Cells*, 1 edn. (Humana Press, 2008).

[25] Dominici M (2006). Minimal criteria for defining multipotent mesenchymal stromal cells. The International Society for Cellular Therapy position statement. Cytotherapy..

[26] Pittenger MF (1999). Multilineage potential of adult human mesenchymal stem cells. Science (New York, NY).

[27] Debacq-Chainiaux F, Erusalimsky JD, Campisi J, Toussaint O (2009). Protocols to detect senescence-associated beta-galactosidase (SA-betagal) activity, a biomarker of senescent cells in culture and in vivo. Nat. Protoc..

[28] Shaffer, L., McGowan-Jordan, J., Schmid, M.. *ISCN 2013: An International System for Human Cytogenetic Nomenclature*. S Karger: Basel, 2013).

[29] Tanaka T (2007). Cytometry of ATM activation and histone H2AX phosphorylation to estimate extent of DNA damage induced by exogenous agents. Cytom. A.

[30] Riccardi C, Nicoletti I (2006). Analysis of apoptosis by propidium iodide staining and flow cytometry. Nat. Protoc..

[31] Kramer A, Green J, Pollard J, Tugendreich S (2014). Causal analysis approaches in Ingenuity Pathway Analysis. Bioinformatics..

[32] Martin A (2010). BisoGenet: a new tool for gene network building, visualization and analysis. BMC Bioinformatics.

[33] Subramanian A (2005). Gene set enrichment analysis: a knowledge-based approach for interpreting genome-wide expression profiles. Proc. Natl Acad. Sci. USA.

[34] Kim JH (2014). Effects of ECM protein mimetics on adhesion and proliferation of chorion derived mesenchymal stem cells. Int. J. Med. Sci..

[35] Lee J (2016). Generation of functional cardiomyocytes from the synoviocytes of patients with rheumatoid arthritis via induced pluripotent stem cells. Sci. Rep..

