# Supplementary material for: Ubiquitin C decrement plays a pivotal role in replicative senescence of bone marrow mesenchymal stromal cells
Source: Cell Death Dis. 2018 Jan 30;9(2):139. doi: 10.1038/s41419-017-0032-5 (PMC5833785; doi:10.1038/s41419-017-0032-5)
Supplement: Supplementary file 1 — Supplemental Information [file 41419_2017_32_MOESM1_ESM.docx]

**Supplemental Information**

**Ubiquitin C Decrement Plays a Pivotal Role in Replicative Senescence of Bone Marrow Mesenchymal Stromal Cells**

Jiyeon Kim^1,*^, Yonggoo Kim^1,2,*^, Hayoung Choi^1^, Ahlm Kwon^1^, Dong Wook Jekarl^1,2^, Seungok Lee^1,2^, Woori Jang^1,2^, Hyojin Chae^1,2^, Jung Rok Kim^1^, Jung Min Kim^3^ & Myungshin Kim^1,2^

^1^Catholic Genetic Laboratory Center, College of Medicine, The Catholic University of Korea, Seoul 06591, Republic of Korea

^2^Department of Laboratory Medicine, College of Medicine, The Catholic University of Korea, Seoul 06591, Republic of Korea

^3^NAR Center, Inc., Daejeon Oriental Hospital of Daejeon University, Daejeon 34520, Republic of Korea

^*^These authors contributed equally to the work.

**SUPPLEMENTARY FIGURES**

**
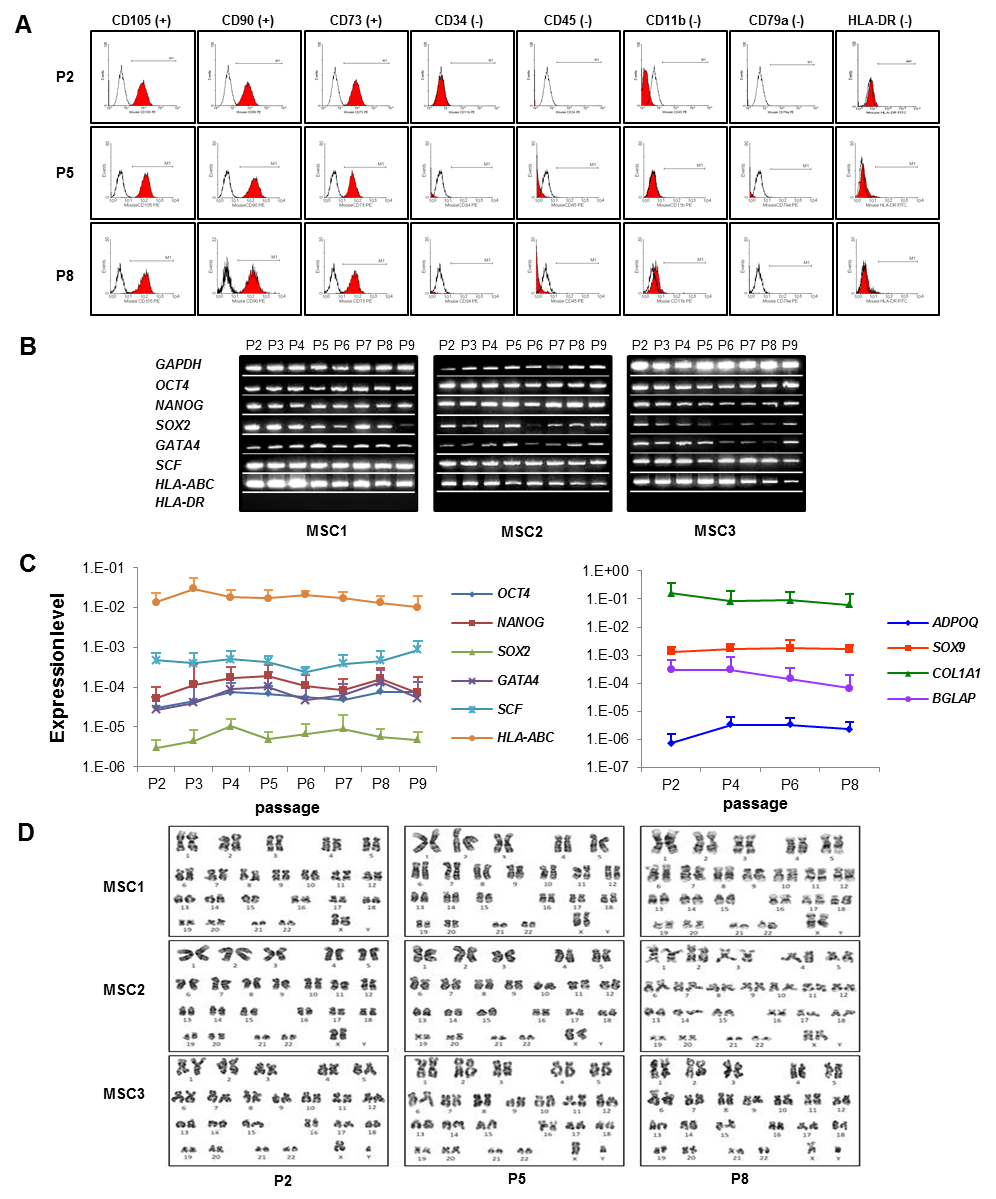
**

**Supplementary Figure 1: Characteristics of hBM-MSCs during *in vitro* culture.**

(A) Immunophenotype analyses of samples P2, P5 and P8 using flow cytometry. Red histograms represent antibody staining and black histograms indicate isotype matched IgG controls.

(B) Gene expression by RT-PCR of stemness markers.

(C) Expression changes of genes associated with stemness and mesodermal differentiation characteristics of MSCs by RT-qPCR during passaging.

(D) Representative karyograms showing normal diploid karyotypes from three donors.


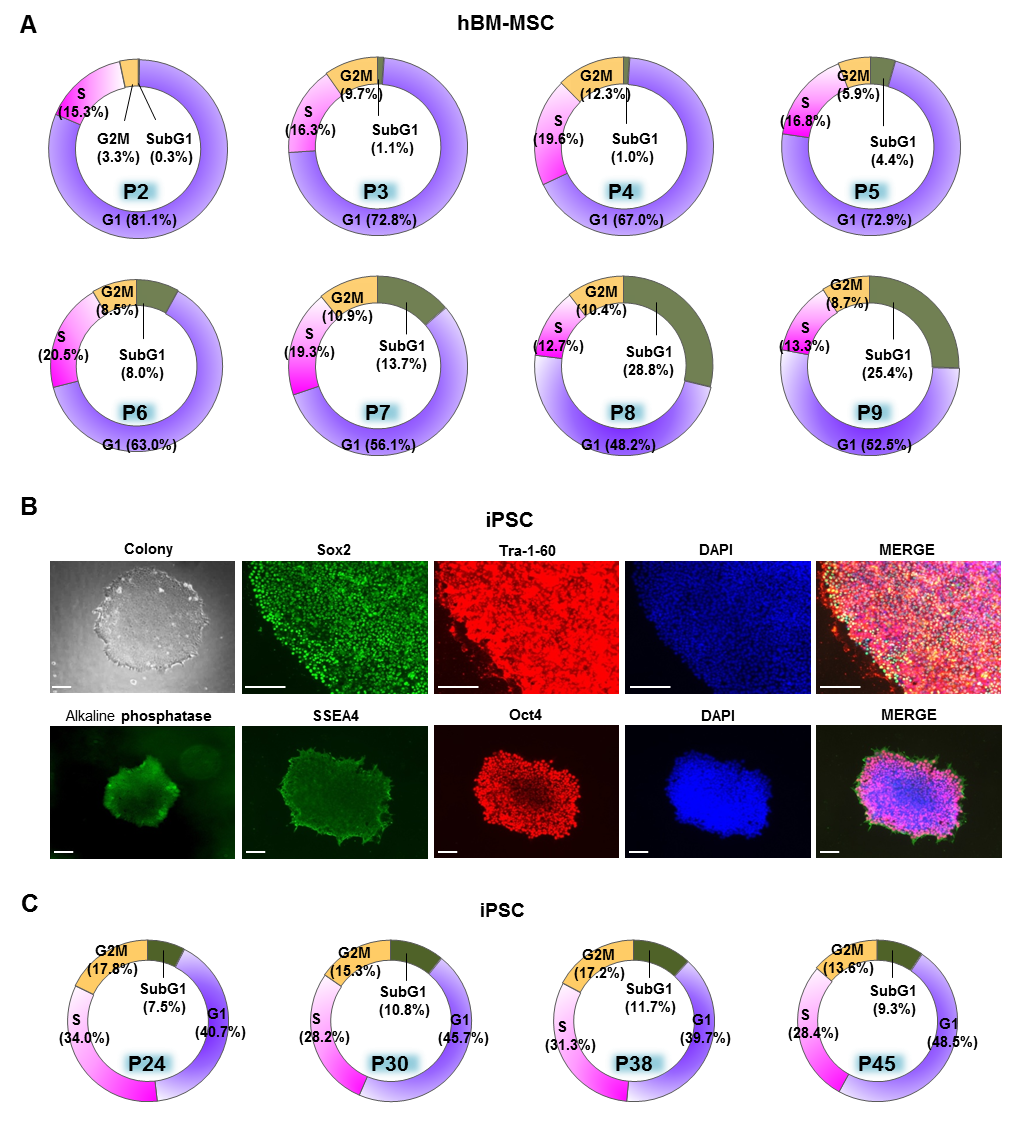


**Supplementary Figure 2:** **Cell cycle changes of hBM-MSCs and iPSCs during *in vitro* culture.**

(A) The hBM-MSC cell cycle during passaging. A significant increase of the sub G1 population during passaging was evident in the three donors.

(B) Identification of iPSCs. Colony morphology and immunofluorescence staining against Sox2, Tra-1-60, alkaline phosphatase, SSEA-4 and Oct4 in iPSCs. Scale bars, 50 μm.

(C) The iPSC cell cycle did not show significant changes during passaging.


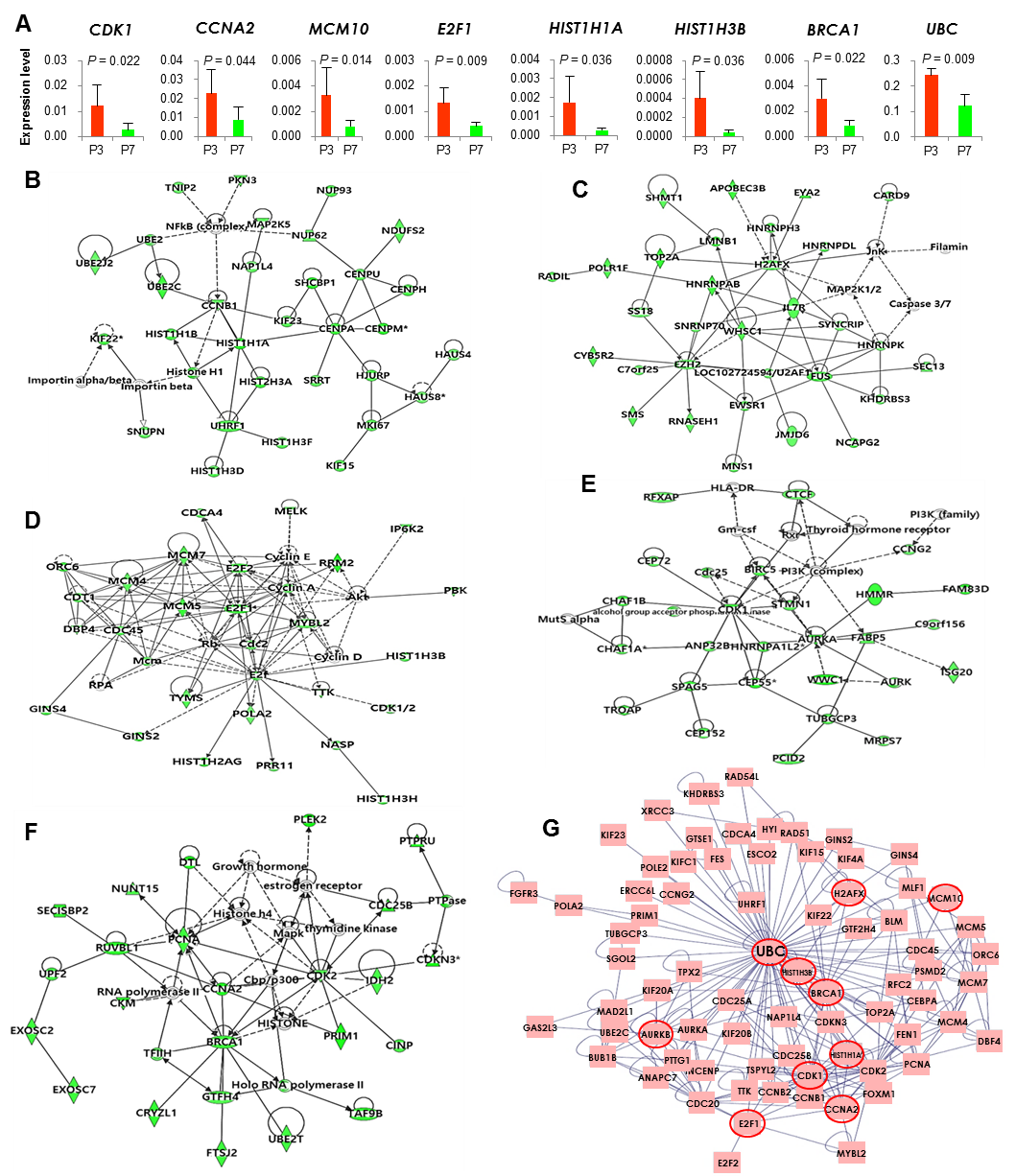


**Supplementary Figure 3: Ingenuity pathway analysis (IPA) network diagrams illustrating annotated interactions between genes affected by *in vitro* culture with the following IPA function.**

(A) Validation of microarray results by real-time quantitative PCR. The results represent mean ± standard deviation of the relative mRNA expression of the selected genes.

(B) Cell Cycle, Cellular Assembly and Organization, Cellular Function and Maintenance.

(C) DNA Replication, Recombination, and Repair, Cellular Assembly.

(D) Cell Cycle, Cellular Assembly and Organization, DNA Replication, Recombination, and Repair.

(E) Organization, Developmental Disorder.

(F) Cancer, Organismal Injury and Abnormalities, Reproductive System Disease.

(G) Gene network from the functional analysis of replicative senescence of hBM-MSCs. Interaction between the significant genes from the functional analysis of downregulated during *in vitro* culture. The 74 genes (pink nodes) interact among each other in a direct way.


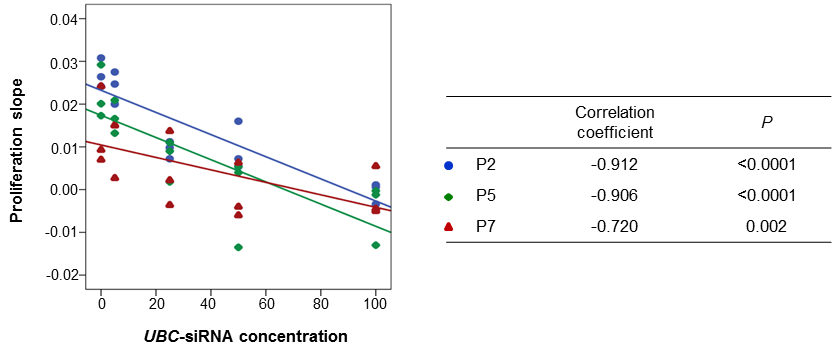


**Supplementary Figure 4: Correlation between *UBC*-siRNA concentration and proliferation activity in *UBC* knockdown experiments.**

**Supplementary Movie:** Live cell image recoding of cell proliferation kinetics of hBM-MSCs after *UBC*-siRNA transfection (right), compared to untreated (left) and negative control (NC)-siRNA transfected (middle) cells. The first record started at 6 hours after transfection with measurement every 15 minutes until 96 hours.

**SUPPLEMENTARY TABLES**

**Supplementary Table S1. Primer sequences for RT-PCR and RT-qPCR**

|  | Genes associated with stemness of MSCs | Product size (bp) |
| --- | --- | --- |
| *GAPDH* | F-ACAACTTTGGTATCGTGGAA | 456 |
|  | R-AAATTCGTTGTCATACCAGG |  |
| *OCT4* | F-CGTGAAGCTGGAGAAGGAGAAGCTG | 245 |
|  | R-CAAGGGCCGCAGCTCACACATGTT |  |
| *NANOG* | F-AGAAGGCCTCAGCACCTAC | 250 |
|  | R-GGCCTGATTGTTCCAGGATT |  |
| *SOX2* | F-GCCGAGTGGAAACTTTTGTC | 264 |
|  | R-GTTCATGTGCGCGTAACTGT |  |
| *GATA4* | F-TTCCCTCTTCCCTCCTCAAAT | 194 |
|  | R-TCAGCGTGTAAAGGCATCTG |  |
| *SCF* | F-ATGCAAATGGTTGCCTGGTCCC | 135 |
|  | R-TGCCAATGTCCTTCTTGGCAGA |  |
| *HLA-ABC* | F-GTATTTCTTCACATCCGTGTCCCG | 394 |
|  | R-GTCCGCCGCGGTCCAAGAGCGCAG |  |
| *HLA-DR* | F-CTGATGAGCGCTCAGGAATCATGG | 220 |
|  | R-GACTTACTTCAGTTTGTGGTGAGGGAAG |  |
|  | Genes associated with mesodermal differentiation | Product size (bp) |
| *ADPOQ* | F-ATGGTCCTGTGATGCTTTGA | 229 |
|  | R-GTTGAGTGCGTATGTTATTT |  |
| *SOX9* | F-TTTCCAAGACACAAACATGA | 112 |
|  | R-AAAGTCCAGTTTCTCGTTGA |  |
| *COL1A1* | F-CCCAGTCCCACCAATCACCT | 119 |
|  | R-CGTCATCGCACAACACCT |  |
| *BGLAP* | F-ACACTCCTCGCCCTATTG | 877 |
|  | R-GATGTGGTCAGCCAACTC |  |

**Supplementary Table S2. Probes for gene expression assay**

| Gene | Assay ID | Reference Sequence |
| --- | --- | --- |
| *GAPDH* | Hs99999905_m1 | NM_002046.5 |
| *CDK1* | Hs00938777_m1 | NM_001786.4 |
| *CCNA2* | Hs00996788_m1 | NM_001237.3 |
| *MCM10* | Hs00960349_m1 | NM_018518.4 |
| *E2F1* | Hs00153451_m1 | NM_005225.2 |
| *HIST1H1A* | Hs00271225_s1 | NM_003525 |
| *HIST1H3B* | Hs00605810_s1 | NM_003530 |
| *BRCA1* | Hs01556193_m1 | NM_007294.3 |
| *UBC* | Hs01871556_s1 | NM_021009.6 |
| *AURKB* | Hs00177782_m1 | NM_001256834.2 |
